# Advocacy, activism, and lobbying: How variations in interpretation affects ability for academia to engage with public policy

**DOI:** 10.1371/journal.pgph.0000034

**Published:** 2022-03-18

**Authors:** Nasreen S. Jessani, Brenton Ling, Carly Babcock, Akshara Valmeekanathan, David R. Holtgrave

**Affiliations:** 1 Center for Evidence Based Health Care, Department of Global Health, Stellenbosch University, Cape Town, South Africa; 2 Department of International Health, Johns Hopkins University, Bloomberg School of Public Health, Baltimore, MD, United States of America; 3 Wholesome Wave, Washington, DC, United States of America; 4 CDC Foundation, Atlanta, Georgia, United States of America; 5 Independent Consultant, India; 6 School of Public Health, University at Albany, Albany, New York, United States of America; University of Milano–Bicocca: Universita degli Studi di Milano-Bicocca, ITALY

## Abstract

Research and teaching are considered core-responsibilities for academic researchers. “Practice” activities however are viewed as ancillary, despite university emphasis on their importance. As funders, governments, and academia address the role of research in social impact, the deliberations on researcher *activism*, *advocacy* and *lobbying* have seen a resurgence. This study explores the perceptions of 52 faculty and 24 government decisionmakers on the roles, responsibilities, and restrictions of an academic to proactively engage in efforts that can be interpreted under these three terms. Data was coded through inductive thematic analysis using Atlas.Ti and a framework approach. We found that discordant perceptions about how much activism, advocacy and lobbying faculty should be engaging in, results from how each term is defined, interpreted, supported and reported by the individuals, the School of Public Health (SPH), and government agencies. Influential faculty factors included: seniority, previous experiences, position within the institution, and being embedded in a research center with an advocacy focus. Faculty views on support for advocacy were often divergent. We surmise therefore, that for effective and mutually beneficial collaboration to occur, academic institutions need to align rhetoric with reality with respect to encouraging modes and support for government engagement. Similarly, government agencies need to provide more flexible modes of engagement. This will contribute to alleviating confusion as well as tension leading to more effective engagement and consequently opportunity for evidence-informed decision making in public health globally.

## Introduction

The relevance of research to practice, policy and the public has been emphasized by funders [1–6], governments [7, 8], decision makers [9, 10] and other scholars in the social sciences [11–14]. This form of ‘engaged scholarship’ [15–19] is notable in policy-focused public health research [20, 21]. With respect to academia, historically, universities were reputed as institutions conducting theoretical, abstract research accessible primarily to the intellectual elite. Over time they have begun to shed this perception by demonstrating emphasis and efforts towards policy and social impact, dissemination and communication of research, through formal institutional vision and mission statements. The changing landscape of academia reflects on the evolving role of a researcher in the field of public health from academia to encompassing activism, advocacy, or lobbying: a researchers that is perhaps more assertively engaged, and perhaps, more impactful.

Policy-focused research includes a wide gamut of topics including policy analysis, implementation evaluation, policy processes and agenda setting etc. Johnston and Plummer [22] distinguish research from advocacy as follows:

***“(1) Research*,** *as generally understood by academics*, *into the existence*, *causes*, *and potential solutions of problems and then*, *if a solution is implemented*, *into its impact;*
***(2) Advocacy*,** *involving arguments to various audiences (and in some cases*, *prior audience identification) regarding the existence and nature of a problem*, *the identification of viable solutions or resolutions*, *and the necessity of political action”*

The intentionality to affect timely and relevant change as a result of policy (or other) research results is key to activism, advocacy and lobbying. However, the interpretation of the terms and the activities associated with them vary.

### Advocacy, activism, lobbying: What do these terms mean and imply?

We begin by outlining how these three terms–advocacy, activism and lobbying—are defined in the purist form. Recognizing that the literature as well as researchers likely come from different Western ideologies of training, we perused British as well as American dictionary definitions as seen in S1 Table.

The various noun and verb definitions of Advocacy, Activism, and Lobbying between the British Oxford English Dictionary (OED) [23] and the American Merriam Webster Dictionary (MWD) [24] indicate subtle differences in meaning within the English language. Both the British and American definitions of advocate are similar with the mention of public support for a cause, person, or policy. The OED further indicates advocacy as a public action. Also, both dictionaries clearly state lobbying as intent to influence a legislator or member of a legislative body. The differences appear starker however in the definition of activist/activism whereby the British OED provides a more political orientation than American MWD, with a specific focus on affecting political change. The consistency between these definitions of expressing active, as opposed to passive pursuit of influence is however notable. There are implications therefore not only in Western organizations but also former colonies in the Global South that encompass meanings and interpretations based on language and historic politics.

As the US government—federal as well as state–increasingly engages in regulating various aspects of the health system, clear definitions of advocacy, lobbying and political activities appear on some of the agency websites [25, 26]. We note that these may at times differ from the pure dictionary meanings and therefore political organizations–as well as academic organizations engaging with them–often provide specificity around their own boundaries surrounding these terms as well as associated activities.

### Current engagement of School of Public Health (SPH) faculty with stakeholders

Research translation and use are increasingly appearing as cornerstone goals across SPHs. SPH faculty however, vary widely in the extent to which they engage with stakeholders–particularly policymakers [27–30] and/or produce research outputs that are timely, relevant and useful [31]. There are several reasons for this: First, the distinction between “pure science” and “applied science” remain entrenched amongst some scientists [32]; Second, the academic incentive structure values research grants, publications, and teaching as currency, with implementation or practice activities often viewed as ancillary [27, 29, 33–42] despite mission statements that prioritize both; Third, the policy environments and the processes for decision-making are complex—involving a multitude of actors, varying timelines and competing political priorities. Navigating this arena is often foreign and unchartered for many faculty. Efforts to engage in this arena that are not transactional or instrumental in nature but instead genuine and relational, appear nebulous, daunting, and dependent on relationships [28, 41, 43–48]. Fourth, engaging on a policy issue is seen by some academics as contradictory to their core endeavors while for others being activist-academics [49, 50] or policy entrepreneurs [51, 52] is central to their role. This institutes a (false) dichotomy between activist and theorist [49, 53, 54] and ultimately results in (self) censorship [15]. This inevitably compromises an SPH’s ability to achieve these aspects of its mission.

The conflict faced by academics in universities has been captured in several studies [55]. These studies highlight that such conflict is laden with (amongst others):

- *Personal* values about the role of researchers,
- *Professional* apprehensions about where to draw the line between communication of research findings and activism so as to preserve one’s credibility as a scientist, and
- *Legal* concerns about where personal and professional boundaries intersect and how that is perceived by the academic institution where one is employed

The various viewpoints amongst academics have argued for both establishment [11, 12, 56] and abolishment [27, 57] of formal structures to support advocacy.

Doberstein [14, 58] found that, at least amongst Canadian decision-makers, that academic research was perceived with and afforded greater credibility than that from think tanks and advocacy organizations. Similarly, Kotcher et al [59] suggest members of the public have expectations of researchers related to engagement. An internal decision-making heuristic using status, expertise and authority as a filter [14] has great implications for academic researchers in terms of privilege, expectations, responsibility and opportunity.

### The SPH response

Regardless of variations in emphasis on advocacy-like engagement, SPHs in the USA are increasingly establishing structures and processes to encourage contributions to public health decision-making. This is apparent in clear guidelines and definitions of activism, advocacy, and lobbying permitted within the Yale SPH [60]. It is also apparent in institutionalized forms such as the establishment of the Activist lab at Boston University SPH where they define an activist as “one that aims to bring about foundational change” and therefore incorporate advocacy training as part of SPH curricula as well as through independent workshops. They also dedicate October as Activist Month.

Commitment to institutionalization of advocacy can be seen through creation of the Center for Public Health Advocacy at Johns Hopkins Bloomberg School of Public Health (JHSPH) [61, 62] as well as the Public Health Advocacy Institute at Northeastern University (although this resides in the school of law). [63] Others, like the UCLA Fielding SPH’s advocacy fellowships, [56, 64] and UC Berkeley SPH and JHSPH’s advocacy courses and certificates [65–67], demonstrate their support through capacity strengthening programs for individuals.

In low- and middle-income countries (LMICs) many of these engagements are embedded in the daily work of academics but not necessarily a focus of the institution’s formal policies [29, 42, 68, 69]. The diverse manifestations of activism, advocacy and even lobbying guidelines and activities across SPHs supports their heterogenous nature. Such endeavors may additionally be underpinned by the lens they apply to (global) public health as one that is technical, humanitarian, social justice centered, entrepreneurial, or some combination [70].

### Aim of this paper

Given the attention to stakeholder engagement, applied scientific practice, and enhanced emphasis on social return on funder investment, we sought to explore the perceptions of faculty at one large SPH, together with the experiences of decisionmakers at the City, State, Federal and Global levels on the roles, responsibilities, and restrictions of an academic to proactively engage in efforts that can be interpreted as activism, advocacy and/or lobbying in the quest to contribute to the policy decision making process. We report on these perceptions, the structural factors associated with them, as well as provide ideas to reconcile the conflict between academic work and activism, advocacy, and lobbying.

We intend for this to paper to provide a direct qualitative report and in-depth analysis of the issues mentioned above; offer suggestions appropriate to leadership of US-based SPHs on actionable steps to help overcome some of the challenges; and provide insight for government decision-makers wishing to engage more with academia.

## Methods

### Context

JHSPH was founded in 1916 and is the largest school of public health in the world comprised of 10 departments, over 70 centers and institutes, and approximately 2200 faculty. Located in Baltimore, JHSPH has unique physical access to city, state and federal government agencies. The school’s primary campus is just 1.5 miles from City Hall, 27 miles from the Maryland State House and just 38 miles from the United States Capitol in Washington DC.

The University as a whole is registered as a private, tax-exempt non-profit organization. For this reason, it is required to report faculty and staff activities that may enter the realm of advocacy and/or lobbying, with restrictions on certain activities. Ensuring that employees understand this has been important to the administration- guidelines for identifying as well as reporting such activities are frequently shared with faculty through email as well as through an online guiding document [71] and website [72]. Furthermore, the Office of Government and community affairs at JHU “works to build and strengthen Johns Hopkins’ relationships with a wide variety of stakeholders”and faculty can contact them with questions through the weblink (http://web.jhu.edu/administration/gca).

At the time of this research, elements of SPH attention to advocacy was established and growing. For instance, JHPSH was offering a course in *Health Advocacy* by the Department of Health Policy and Management [73] and one on *Media Advocacy and Public health* by the Department of Health Behavior and Society [74]. Faculty publications of advocacy relevant topics were also diverse ranging from informing policy debates on critical issues such as injury [75] and mental illness and gun policy [76], to the role of public health professionals in informing the judiciary [77], to implications for public health education and curricula [78–80].

Focused attention to advocacy within programs relevant to family and reproductive health was also apparent through the Gates Foundation launch of the Advance Family Planning (AFP) advocacy initiative at JHSPH in 2009 [81]. Furthermore, interest in understanding how advocacy manifests at JHSPH and its implications was explored in 2013 and led to recommendations for a high-level forum on public health advocacy [82]. In this white paper, Rimon et al [82] define advocacy as follows: “Advocacy aims to influence policy and practices in ways that benefit people’s health and well-being and the societies in which they live. Advocates within government, civil society and academia use evidence and other rationales to improve the social good by:

- encouraging positive changes to the law, and to government and service policies;
- improving access to scientific data and evidence;
- increasing financial support for interventions that improve health; and
- altering or shifting public attitudes and behaviors.”

This study therefore complements and expands on these initial internal explorations to provide empirical evidence—from within the JHSPH but also externally from decision-makers who engage with the SPH—on implications for public health practice.

### Participant selection, data collection and analysis

Our initial study population consisted of 211 full-time faculty from JHSPH yielding relationship with over 700 decision-makers at the Baltimore city, Maryland state, USA federal, and global levels [28]. Decision-makers were defined as those individuals in a position to make decisions or exert influence in a decision-making situation and who plays a key role in the administration or leadership of an organization.

As with previous mixed-method studies [29, 83, 84], ours consisted of a combination of quantitative and qualitative data collection methods. The initial phase of our study was conducted between May–December of 2016 and sociometric surveys were used to map academic faculty and decision-maker networks. In establishing faculty decision-maker network maps, we sought to determine what factors were involved in building and maintaining relationships by performing in-depth interviews with a subsample of faculty and decision-makers. The faculty that were selected for interviews met the following inclusion criteria: Highly engaged—Faculty who had 5 or more contacts with decision-makers at any one government level and/or in the top 10 percentile of those with the most connections across all four government levels (n = 49); Non-engaged- Faculty with 0 or 1 contacts with decision-makers (n = 57). Inclusion criteria for decision-makers focused on those who were mentioned by faculty on 2 or more occasions (n = 92).

The semi-structured interview (SSI) guides were adapted from a previous study [29, 85] and reviewed by field experts for accuracy. Socio-demographic information was collected including age, gender, academic qualifications, and some organizational information (departmental affiliation, leadership position, academic position) in order to contextualize any variation in responses.

There were no direct questions on activism, advocacy or lobbying but opinions on these emerged organically when asked questions about the nature of engagement between faculty and decision-makers -more specifically how faculty became involved with a particular issue and how evidence was promoted or shared with decision-makers, and ways in which JHSPH could provide training for faculty with these engagements. For decision-makers questions that explored the role of researchers in bringing evidence to bear on the decision-making process, and potential facilitators and barriers to evidence-informed decision-making elicited responses relevant to activism, advocacy and lobbying.

Eligible faculty and decision-makers were contacted a maximum of twice for interviews occurring between November 1, 2017 to February 5, 2018 either in-person, over video Skype, or by telephone. Interviews typically lasted between 30–75 minutes depending on respondent availability and were audio-recorded with participant verbal consent. Participant responses were transcribed verbatim, and all transcripts were imported into ATLAS.ti [86] for analysis. A common codebook was developed to inform thematic analysis; a sample of transcripts were co-coded by the study team and reviewed as a group, in order to establish inter-coder reliability while remaining transcripts were coded individually.

Codes were extracted for content and multiple responses for a single participant were then consolidated in Excel using the framework method for analysis [87]. Respondent quotes were extracted using deductive thematic analysis and coded based on predetermined categories associated with interview questions. Following initial analysis overarching themes emerged from which inductive thematic analysis resulted in a number of subthemes that centered around advocacy activities. Since these were unanticipated, in addition to inductive thematic analysis transcripts were additionally searched for the terms ‘advocacy’, ‘lobbying’, and ‘activism’ in order to collate those quotes appropriate for additional thematic analysis. The resulting quotes painted a robust picture of the complex nature of advocacy and its role or place in faculty decision-maker engagement.

Ethics approval for the study was granted by the Johns Hopkins Bloomberg School of Public Health Institutional Review Board.

## Results

An overview of the 52 faculty participants can be found in S2 Table. Of the 92 decision-makers eligible from Phase I, only 69 were still in their original roles or with the organization during phase II. Of those 24 participated in the study with the following distribution: City—6, State– 9, Federal– 3, and Global– 6.

We report on the dominant themes that appeared from our interviews which include how the various terms are perceived; differences across government levels; the role of academics in advocacy; and examples of advocacy activities (direct as well as indirect).

### Definitions, interpretations, and how they manifest in reality

The recognition of engagement being driven by the goal of influencing an outcome was expressed consistently by decision-makers and embedded in their stories of initiation and motivation behind partnerships: *“Any proactive form of engagement and trying to communicate that value of a piece of science for public health purpose (is welcomed)*.*” (Decisionmaker_Global_1)*

While we did not ask questions specific to advocacy, activism, and lobbying in our interviews, faculty members often responded to our questions about faculty/decision-maker engagement by raising notions of one or more of these. However, utilization of the terms often deviated from the official definitions of these and were used more colloquially. For example, the term “lobbying” was sometimes used to describe efforts to influence people and issues far beyond the political parameter set by the dictionary definition—using it to describe efforts to influence non-politician or legislative bodies such as university leadership for a range of things from additional resources to new policies.

One faculty member acknowledged the lack of clarity around the definition of lobbying–whether it meant trying to influence decision-making with research–stating: *“We could have several lengthy arguments about whether providing scientific advice is lobbying” (Professor_1)*.

Decision-makers provided different interpretations of the rules that govern lobbying at various jurisdictions, particularly when recounting their experiences with faculty running against the rules for lobbying. We note this in the first quote below by a City government official, which was supported by a response from a faculty member:

*“Lobbying—That needs to be addressed at your (academics’) end…Are you working under some grant or other restraint against lobbying*? *And what does that mean*? *I mean lobbying could mean a lot of things*. *It could mean political lobbying—that is not technical lobbying in our terms*. *Lobbying means communicating with the legislative body to seek change and you are expending or spending salary payment for that and that could be internal even if you are an employee*.*” (Decisionmaker_City_1)*.

“*I sit on one of the study section panels that reviews grants for (The National Institute of) General Medical Sciences (GMS) and we are instructed that we do not review a grant in translation to something bigger*. *We review it on its science*, *its discovery*. *There’s a whole part of (NIH) that is devoted to new science*, *and I would say that maybe 60–70% of our money comes from GMS” (Professor_4)*.

A breakdown in engagement when there was a misunderstanding of the guidelines and requirements for registering as a lobbyist is described below:

*“Many folks in academia are not aware of the fact that they more than likely need to register as lobbyists for what they are doing and that became problem with one group*. *I think the Bill was introduced …and there was a brouhaha as they (the academic colleague) hadn’t registered as a lobbyist—and the reason I think was that they were pursuing or working under a grant that expressly indicated that they can’t engage in lobbying*, *or however it was expressed in the grant*. *Therefore*, *they might have lost the grant if they did register and that became a problem” (Decisionmaker_City_1)*.

Amongst the three terms “lobbyist” when used in its noun form was the clearest and least contested due to its political underpinning despite inconsistencies on who was target audience. However, in its verb form, it was often used interchangeably with the verb “to advocate.” Activities therefore were more favorably viewed that labels: ie doing advocacy work was different than being called an advocate. The terms “activist” and “activism” appeared more rarely.

### Differences across government levels

Faculty respondents were acutely aware of the differences in engaging with various levels of government, both in terms of mission as well as process. In one such example, a faculty member noted that the “lower” levels were focused on solving high-urgency health problems while the “higher” levels were focused on writing longer term policy improvements. However, there was little discussion or mention of the uniqueness to global policy advocacy.

Faculty as well as city decisionmakers were well aware that the City government tends to have fewer resources at its disposal, particularly for research. The onus, therefore, of seeking out what types of information City officials need to improve health-related policy appears to rest on the research community. Faculty felt that that city-level decision-makers were, in several cases, more accessible than state, federal or global policy decision-makers and therefore this responsibility didn’t appear too contentious.

### The role of academics in advocacy (whether for policy or practice)

#### The role of the individual

Regardless of the level of government that faculty engaged with, we found a variety of perspectives on whether advocacy falls under the remit of researchers. Most faculty expressed that the active pursuit of engagement with decision-makers for the purposes of influencing decisions was an inherent responsibility or duty of an academic. *“I think it’s always good to be an advocate for what your department is doing*, *what you are doing*, *what you can bring to the table and you need to be a voice as you can’t expect people to always reach out*.*” (Assistant_Professor_1)*.

However, the extent to that engagement and its appearance as advocacy was met with mixed perspectives:

“*I think most researchers are still scientifically minded and believe research should be scientific at their core and not be influenced by policy or policymakers and believe researcher’s job is to do the science*, *provide the evidence and somebody else should take the baton and pass it along*.*” (Assistant_Professor_1)*.

*“There is a general feeling that doing advocacy is kind of like doing a four-letter word act in public*. *You’re somehow “tainted” if you’re an advocate*. *I believe there are many people who subscribe to that philosophy*. *They feel research is a pure thing*, *and the only way to advance is to do really good research*. *I obviously don’t agree with that*. *It’s just not correct*, *although there are many people who feel that way*.*” (Associate_Professor_1)*.

While extremely reluctant to “speak for others”, faculty respondents offered hypothetical contrary positions to their own. *“…obviously different areas of research lend themselves more to policy advocacy than others” (Professor_2)*. In addition, several faculty did note that one’s career can still be successful even in the absence of engaging in advocacy, and in some cases, even necessary. However, those who subscribe to the importance of advocacy posit that careers should not be at risk since: *“I think [the University] is big enough to allow some to do this [advocacy]*, *and some to do that [no advocacy]*.*” (Professor_3)*.

Decisionmaker respondents did not express a clear opinion either in support of or in opposition topolicy engagement being an inherent responsibility of academics. However, several of these respondents shared that academics engaging with intention was becoming increasingly more common. *“…we cannot generalize- but we see more and more certain academic groups being more concerned about the implications of their work*.*” (Decisionmaker_City_2)*. One decisionmaker noted that rules around registering as a lobbyist were different based on city or county level, and they found that restrictions on academics to register as a lobbyist in one city to be a limiting factor for their ability to influence policy.

While academics described their decisionmaker engagements and their willingness to publicly advocate for certain policy or practice influence, they also shared concerns about the need to maintain an unbiased and/or neutral reputation. *“There is always a concern of being an impartial scientist and not being objective*.*” (Assistant_Scientist_1)*. This was echoed by decisionmakers, *“Actually when we work with experts*, *we ask for declaration of interests and we don’t engage with researchers who will gain benefits from the policy area–it could be both financial or their own academic interests*. *Ensuring scientific integrity and rigor*.*” (Decisionmaker_Global_1)*.

#### The role of the institution

In order for faculty to engage in advocacy activities, the role of the institution was also raised. Decision-makers appeared to appreciate efforts made from the SPH, in contrast to individual faculty, to engage with them on key matters pertaining to policy and practice decision-making. However, one decision-maker indicated wanting to see more of this.

From the faculty perspective, support from leadership on engaging in advocacy activities was recognized and welcomed: *“I appreciate that management understands that advocacy is important*. *Because in public health*, *if you aren’t trying to change things for the better you aren’t really fulfilling your goals as a health professional*.*” (Associate_Professor_1)*.

Opinions on this support began to diverge, however, when there was a perception of ‘politicization’ or ‘disabled neutrality’- oftentimes occurring in parallel with word choice. For instance, if the word “lobbying” was used in conjunction with or as replacement for “activism” or “advocacy”, perceptions of leadership support altered. The interpretation of “lobbying” clearly varied amongst faculty with concerns that perhaps how faculty interpret relevant activities may not align with guidance from the SPH: *“We are very concerned about lobbying*, *we are very concerned with use of the Johns Hopkins name*, *and all of that becomes a little intimidating for some faculty who really get the message of against talking to the policymakers because it could interfere with the Johns Hopkins message that they’re trying to convey*.*” (Professor_3)*

Faculty noted that the rules for visits to the State and Federal legislature came with increased scrutiny as faculty members were required to report these to the university. *“I’ve seen faculty reprimanded for jumping past the lobbyists and representing the university*.*” (Professor_4)*. This is likely justified in most cases since faculty are generally permitted to speak in their individual capacities but not as representatives of the university, a regulation that is common across most institutions. One respondent indicated that while they empathize with the SPH’s concerns from a legal standpoint, this hinders the cause of research informing action.

### Direct advocacy: Capacity, skills and experience

Faculty willingness and likelihood to engage with decision-makers and advocate for change in a meaningful way was sometimes inhibited by their ability to do so. Several respondents expressed that a significant barrier to advocacy was simply a discomfort with the skills and traits needed to be a successful advocate. *“I don’t know the first thing on (advocating for educational tax exemptions) and I would love some training for doing that*.*” (Assistant_Scientist_2)*. Many faculty respondents expressed an interest in advocacy training, which included skills such as problem identification, power mapping, coalition building and communications. Additionally, some noted that the school itself had the skills in house to create advocacy resources for current academics. Others suggested increasing the emphasis on advocacy skills as a part of graduate school curriculum.

Faculty also noted that having more seniority within the school was an influential factor: *“I think some of the advocacy rulings of the university are constraining for young faculty who are developing their career paths…*.*” (Professor_6)*. However, another junior faculty member recognized that having access to the school’s senior researchers, and the reputation they carry could also facilitate advocacy opportunities for them, *“[This] is a leading SPH and so the researchers*, *especially senior researchers are some if not the best in the world*. *(Name redacted) has been working in this issue for 30 years so has a wealth of knowledge; unlike me who is only in (his/her) 30s*.*” (Research_Associate_1)*

### Indirect advocacy: Leveraging intermediaries

#### Distinguishing the personal from the professional

Oftentimes, forms of indirect advocacy were viewed in a positive light in contrast to more direct forms of advocacy. For instance, some faculty members expressed being more comfortable participating in general advocacy for broad categorical issues like funding for public health activities, while remaining a safer distance away from taking a position on specific pieces of legislation. Others said they join nonprofit associations that fund advocacy work they agree with and use opportunities like “lobbying days” organized by those association bodies to participate. Structures such as these offer a space for personal engagements with an independent association to be clearly distinguished from professional engagements perceived to be associated with the SPH.

#### Leveraging advocacy organizations

One way around the concern about risking reputation, neutrality and potentially even university rules was to tailor some of the research outputs in ways that they could be used by professional advocacy organizations. These strategies ranged from looking ahead to policy opportunities on the horizon and tailoring research questions appropriately, to writing up summaries of research findings in a format usable by advocacy organizations (i.e. a policy brief): *“So*, *while I feed information and partner with advocacy groups*, *I don’t have conversations with a policymaker*. *It just stops at like a factsheet*, *a training*, *something like that*. *I certainly do promote my work with nonacademic stakeholders*.*” (Assistant_Scientist_1)*.

Professional associations like the American Public Health Association were also reported to play a similar role of facilitation due to their ability to hire third-party expertise for advocacy efforts. However, faculty noted that these efforts were further from members’ control due to the vast and broad membership structure of these types of associations. Their value toward advocacy was also noted by a decision-maker respondent *“Civil Service Organizations have an important role to play either as a lobbyist or driver (toward evidence informed decision making) for an academic” (Decisionmaker_Global_2)*.

#### Leveraging University-based Research Centers (URCs)

Another idea revolved around creating and/or leveraging dedicated roles for activism and advocacy. For instance, University-based Research Centers (URCs) or Institutes at JHU provide a platform for advocacy activities: *“the (decisionmakers) all came to the table to talk about our center and our work and (center director) is leading that relationship between those individuals to try to push along some legislation*: *trying to decide when (they) go to the Hill (sic) with these issues*, *what exactly should they propose etc…*.*” (Research_Associate_1)*. Many such URCs have advocacy as part of their mission thereby legitimizing such activities and permitting employment of dedicated staff for information dissemination or advocacy.

A handful of respondents expressed that such centralized entities could play an important role in the process of faculty and policymaker engagement through their ability and expertise to advocate or lobby. This was noted as the case for the Moore Center for the Prevention of Child Sexual Abuse, and the Center for a Livable Future. The Center for Alternatives to Animal Testing noted that *“…there is an MOU between the University of Konstanz in Germany and JHSPH—where there is a full time person in the European Parliament who is registered as a Lobbyist…used to have the same on the US side…” (Professor_1)*.

#### Leveraging funders

Other intermediaries with particularly significant influence in the research and engagement process are funders. Respondents shared examples where funders increasingly pushed for research outcomes over outputs, while others noted that recent cuts in USA federal spending on research heightened the need to both demonstrate impacts of research as well as continue advocating for renewed investment.

*“Is there funding that can include or expect engagement with policymakers*? *Yes*, *that’s all my finding*. *That’s what my program is*. *I’m going to take the science to Capitol Hill*, *I’m going to work with advocacy groups*, *I’m going to develop some of the science*. *I’m going to bring that into teaching*. *I’m going to do outreach*, *etc” (Associate_Professor_1)*.

*“But also depending on the funder*, *funding mechanisms can make it possible to create relationships with policy makers so you would naturally be in contact with them*. *For example*, *in these UNICEF and USAID funded projects you may be engaging with a minister of health or more often at a lower level than the minister …” (Assistant_Professor_2)*.

However, government funding was also repeatedly cited to have strict restrictions on lobbying or advocacy activities associated with the grant, *“what advocacy can I really do if I am NIH funded*?*” (Assistant_Professor_3)*.

Federal as well as Global decision-makers in our respondent pool often played the role of funder, thereby steering the research to questions more applicable to anticipated and relevant policy and practice decisions. In such cases, decision-makers themselves play the role of advocates using evidence to demonstrate support for or against a policy decision. This process does not only relieve the responsibility for advocacy from the academic but may also be an opportunity for an indirect activist to enter into engagements that will advance EIDM.

There was concern however that funding research for the intention of policy and practice influence could misguide research that otherwise may have been more important to public health.

#### Decision-maker perspectives

From the decision-makers’ perspective, there appeared to be concurrence about the potential role of intermediaries. Some suggested that there may indeed be better presenters of research results and their implications than the academics themselves, describing helpful interactions with such actors. Whereas others expressed that this translation was precisely the role of academics due to their credibility and expertise: *“When it’s time to testify*, *we always bring JHU in*. *They are included in the whole process*, *but we write our own briefs and remarks and presentations based on the data (that JHU provides)*.*” (Decisionmaker_City_3)*.

This may be due to concerns of misinterpretation or misunderstanding of the content by a communications or lobbyist third party, as expressed by a City-Level government decision-maker here:

*“We had to think about how we protect the scientific definition […] so that they couldn’t change our research and that is absolutely essential*. *If I have scientific evidence that could be vulnerable to legislation*, *I would put a layer on top of it and change the name slightly so that is what gets changed in legislation rather than the actual research itself*.*” (Decisionmaker_City_3)*.

In order to avoid such misinterpretations and ensure a holistic understanding of the evidence, one decision-maker reported to have collaborated with intermediaries or third parties to scan, validate, and surmise research outputs beyond a single academic engagement.

## Discussion

As academia traverses from generating purely scientific impact to additional social and policy impact, the roles of researchers, decision-makers and the citizens in the research-to-impact interface is also evolving. However, the path to impact can vary from dispassionate dissemination of research evidence to more engaged advocacy. SPHs promote faculty and research impact through mechanisms such as translation of research results into public policy. However, at what point does this comprise activism, advocacy or lobbying? For those who enter the field of public health to have an impact on the populations they serve—and with school missions echoing this same notion—it can seem counterintuitive and even confusing to be both, encouraged to influence policy while having mechanisms that prohibit influencing politics, when the two are so intertwined.

We studied the perceptions of such activities with academic faculty at one SPH as well as those of decision-makers from the City, State, Federal and global agencies who engage with the SPH. This was against the backdrop of studies that and perceptions capturing conflicting support, encouragement, resistance and distaste for activities comprising these terms across a variety of stakeholders in the research to policy and practice interface.

We focus on two ideas in this section: The first is what we know and how to think about academics as activists, advocates and lobbyists. The second summarizes lessons for scholars, administrators and higher education leadership who are attempting to align the practices of their faculty with the missions of the organization.

### A is for…?

While activism, advocacy and lobbying are components of the larger movement on engagement with decision-makers (stakeholder engagement, integrated knowledge translation, co-production of research etc.) they do not present the same benefits or challenges as other activities that fall under the larger umbrella given the contentious nature of their interpretation. Our results indicate that discordant perceptions about how much activism, advocacy and lobbying faculty should be engaging in, results from how each term is defined, interpreted and reported by the individuals, the SPH and government agencies.

Personal values, professional norms, and institutional regulations across contexts vary greatly, presenting heterogeneity in where academics lie on what can be described as a continuum of activism [88, 89] regardless of how they self-identify, given some commonalities inherent to their pursuit of socially relevant research [53]. This may be compounded by differences in even purist definitions of these terms across British and American dictionaries that imply cultural embeddedness in how these activities are perceived and encouraged. It is therefore not surprising that we see diverging opinions in how SPH faculty perceive, judge and engage in advocacy activities. It is also not surprising that these opinions result in either implicit and unrecognized activism, explicit but downplayed advocacy, or explicit and celebrated strategies.

Similar to studies from the UK [55], our study suggests that these interpretations conceptualize advocacy as either “representational” or “facilitational” [90]. There is a danger that ‘benign and acceptable’ activities currently supporting evidence informed decisions may suffer rejection or retribution if indeed they are officially recognized as advocacy. Without definitional clarity, the debates and confusion are likely to persist. We therefore recommend dedicated attention to providing this clarity and ensuring that faculty and partners alike have a clear understanding of the activities that fall within each.

Scholars studying the challenges of academic engagement with decision-makers have garnered experiences from several contexts to provide guidance on strategies for engagement [29, 91–93] as well as tools to support enhanced use of research findings [94–96]. However, any academic endeavor in advocacy training would need to be coupled with experiential opportunities if deep learning and true competency is desired [56], else, theory is unlikely to translate into practice. Some programs that attempt to address this goal include The Science and Technology Policy Fellowship [97], and the Health Policy Fellowship [98]. The results of the current study suggests that HEIs and programs would benefit from additional translational partnerships with relevant academic entities.

It would be remiss not to recognize the impact of the recent COVID-19 Pandemic which has resulted in sweeping attention to public health, public health experts and SPHs globally. We have seen several academics previously not used to public engagement being requested to provide their thoughts, opinions and advice thereby throwing them into the public eye. It is likely that previous established professional connections catalysed some experts receiving more requests than others.

As one faculty member at a UK-based University reported in an academic professional news publication “*the challenge of COVID-19 has produced a range of new and productive relationships between the academic and policy worlds*. *It has demonstrated the huge potential for bringing together government with a much wider range of disciplines than has traditionally been the case*. *But the future of these relationships is far from assured*” [99].

The above may have provided encouragement for some researchers to engage with—or caused others to shirk further away from—the public eye during the pandemic. Regardless of the effect on academics, what the pandemic has shown is that evidence-informed decision making is critical and that evidence from academia is considered rigorous, trustworthy and relevant in times such as these. It has also shown that skills to engage with the public and in advocacy are critical to advance changes in policy, practice as well as public attitudes and knowledge.

### Implications for researchers, scholars and academic faculty

For scholars who explicitly identify as activist-academics [49, 50] or policy entrepreneurs [51, 52] there is a recognition that their research interests align with their political values and beliefs [100] resulting in advocacy being entrenched in their very approach to research. For such faculty, direct advocacy doesn’t present much discomfort. However, understanding whether they should expect indifference, support or discouragement from the administration can present anxiety.

For academics struggling with the cognitive dissonance of remaining a neutral academic while still trying to affect policy or practice change, one option would be to engage in indirect advocacy. This could be by aligning professionally with an epistemic community that contributes to the actions of an advocacy coalition [101] or more personally with a social movement. Weible et al [101] highlight the differences between different types of political associations–in particular, advocacy coalitions—championed by “policy actors” trying to influence policy—and epistemic communities–championed by experts trying to influence policy. The intensity and length of time of such associations may be determined by the nature of the policy problem in terms of its threat (eg: chronic, acute, sporadic,) its temporality (eg: sudden, enduring, etc) and in its likely affect (for example, coalition formation and/or maintenance) [101] thereby permitting academics to engage in different ways. Such associations serve as intermediaries or knowledge brokers between the research and policy community. Other intermediaries that can also serve a brokering function include funders, the media, consortia [102], and knowledge translation platforms–particularly in LMICs [103–106].

Public health as a field often involves public health policy and programmatic studies that are essential to its evidence base and its relevance. Because policy studies and relevance are so critical to public health, one might posit that there are more such instances of interactions between public health academics and policy and programmatic partners in government. While this can certainly occur in medicine and law, perhaps public health has an especially close nexus to policy making relevance. Regardless of how academics choose to engage and where on the continuum they lie, we recommend reflective scholarship as critical for enhanced contribution to change [107, 108]. This reflexivity and positionality, we argue, extends to SPHs as well.

### Implications for SPHs and other Higher Education Institutes (HEIs) globally

Reflection on institutional opportunities, policies and processes for faculty advancement and promotion need to occur to recognize, support and reward efforts and activities that promote engagement with stakeholders with the aim to influence change in policy and/or practices [109–113]. We assert that these need to be cognizant of the varied foci of the various departments within an SPH as well as the predilections and seniority of faculty. This may also vary across SPHs that are embedded in universities or HEIs that bear different legal status’ (eg private vs public, for profit vs not-for-profit etc). Guidance however, on how to navigate this space can be found through several organizations [26, 114, 115]

Given that definitions vary between British and American dictionaries, we posit that these terms manifest differently across faculty regardless of training, or across practice of activities that fall under these definitions in different parts of the world. We urge SPHs such as JHSPH that comprise international faculty and engage with stakeholders globally, to seek to clarify these terms, identify permissible and prohibited activities that fall within them, and ensure that all staff are aware of these. Furthermore, we recommend that these be accessible to all stakeholders that the SPH engages with in order to avoid confusion and manage expectations regarding the extent to which faculty can engage, particularly with respect to policy decision-making. One question remains however: if the formal rules on lobbying are limited to legislatures, what does that mean for engagement with administrative / agency officials? We call on SPHs to facilitate this discussion further.

### Advancing advocacy at JHSPH

Advocacy has increasingly become important at JHSPH since the beginning of this study. In 2018 the strategic plan for JHSPH highlighted advocacy among the goals set forth in order to ‘set the best course for the school’s future and unleash the power of public health’ [116]. More specifically, it urges faculty to go beyond scientific publications and become better at engaging decision-makers and others of the importance of evidence-informed programs and policy. With a core strategy of the school over the next four years being one of advocacy, the launch of the Center for Public Health Advocacy housed within the Department of Population, Family and Reproductive Health, is well situated to ‘support a culture of evidence-based advocacy’ [62] and enhance faculty capacity to reach this goal. We have seen the advent of more courses being offered such as *Policy Advocacy in Low- and Middle-Income Countries* through the Department of International Health’s Summer Institute [67], thereby implicating yet another department at the SPH in the quest for capacity strengthening in advocacy. Furthermore, a grant from Bloomberg Philanthropies provides support for Science communication and advocacy through the Bloomberg Health Initiative [117, 118].

The implications of nuanced terminology and its interpretation in practice at the local as well as the global level should be of primary interest for an internationally reputed institution such as JHSPH that seeks to build the next generation of (culturally) competent public health researchers and faculty at the local, national and global levels. Knowledge, skills and enhanced faculty competence in advocacy therefore becomes critical for the SPH.

### Strengths and limitations

This is the first study that we are aware of that has unpacked the perceptions of public health academics as well as government officials on the notions of activism, advocacy and lobbying in a changing political and funding landscape. The use of different modes for our interviews (video Skype, telephone) allowed us to expand reach to global decision-makers and those not located in Baltimore, MD. The qualitative data collection process elicited increased reflections on a topic that some faculty (especially non-engaged faculty/faculty from basic science departments) had not had the opportunity to do before.

However, given that discussions about activism, advocacy and lobbying emerged organically from the interviews we may have missed out on some interesting perspectives had this been a deliberate question. In terms of our anticipated sample, changes in the political administration in the USA in 2016 led to large scale turnover of political appointees and government officials. This turnover resulted in a shorter list of invitations being sent (69 decision-makers rather than the eligible 92 from Phase I of this study).

We acknowledge that female academics, junior colleagues, and academics of color may have different experiences in translational work. However, given the small numbers of interviewees within each of these groups as well as the multi-factoral diversity of our respondents suggests that the experiences vary greatly based on the attributes above but also in terms of support, values, previous training, former experiences, and other factors. Those interviewed may not therefore be representative of all faculty at JHSPH therefore cautioning against generalizations among persons of any sociodemographic group. Furthermore, providing such descriptors against some of the quotes would likely reveal, in some cases, who the respondent is and there might be the possibility of respondent identification. For these reasons, comparison between respondents in terms of gender, race, ethnicity and career length requires further research and a much larger study than was possible in the current work. We believe that such expansion of this line of inquiry is indeed important to better understand and build health equity.

Lastly, the changing public health landscape as well as the change in government in the USA in the last 18 months might evoke different responses from our participants than what we heard at the time of this study. We have witnessed policy decision-makers reaching out more to researchers, we have observed the reaction of the public to the politics of the science, and we have experienced the powerful role of the media in contextualizing scientific expertise. We have also noted professionals in the field receiving glory as well as abuse for their contributions, and funding for public health has grown steeply in the face of the COVID-19 pandemic.

## Conclusion

We present arguments as well as some recommendations on how researchers and SPHs in particular, can address the complexity of what research impact might imply. While there are some clear recommendations that we have suggested, the approach to addressing how an academic institution engages with influencing social and policy deliberations will likely be constrained by its context, its value system, and its leadership. This is likely to vary within as well as between countries that have complex political and funding influences. Studies analyzing the proclivity of SPHs to address their relationships with activism, advocacy and lobbying taking the larger contextual issues into consideration would help connect some of these arguments and recommendations to practical considerations. Furthermore, comparing perspectives across research institutions might provide more insight into the role of resources, global status, reputation, geographic location and connections. Additionally, it might be interesting for future research to explore and compare perspectives on activism, advocacy and lobbying between US academics and those in other parts of the world.

## Supporting information

S1 Table British and American dictionary definitions of terms. (DOCX)

S2 Table Faculty participant overview. (DOCX)

S1 File Interview guide for faculty. (PDF)

S2 File Interview guide for decisionmakers. (PDF)

## References

[1] UKRI. Pathways to Impact. 2018; Available at: https://www.ukri.org/innovation/excellence-with-impact/pathways-to-impact/. Accessed May, 2019.

[2] NERC. Innovation Follow-on Call: Enabling innovation in the UK and developing countries—Announcement of Opportunity. 2017; Available at: https://nerc.ukri.org/innovation/together/opportunities/. Accessed May, 2019.

[3] AHRC. Arts and Humanities Council (AHRC) Follow-on Funding for Impact and Engagement. 2018; Available at: http://www.fundit.fr/en/calls/ahrc-follow-funding-impact-and-engagement. Accessed May 209.

[4] Warry P. Increasing the economic impact of research councils: advice to the Director General of Science and Innovation. DTI from the Research Council Economic Impact Group. 2006;06/1678.

[5] McLean RKD, Graham ID, Tetroe JM, Volmink JA. Translating research into action: an international study of the role of research funders. Health Research Policy and Systems 2018;16(44):1–15. 29793541 10.1186/s12961-018-0316-y PMC5968540

[6] Smits PA, Denis JL. How research funding agencies support science integration into policy and practice: an international overview. Implementation Science 2014;9(1):28. doi: 10.1186/1748-5908-9-28 24565209 PMC3939639

[7] HEFCE. Research excellence framework: second consultation on the assessment and funding of research. 2009.

[8] Government of South Africa. White Paper on Science, Technology and Innovation. 2019.

[9] Oliver K, Innvær S, Lorenc T, Woodman J, Thomas J. A systematic review of barriers to and facilitators of the use of evidence by policymakers. BMC health services research 2014;14(1):2. doi: 10.1186/1472-6963-14-2 24383766 PMC3909454

[10] Dobbins M, Rosenbaum P, Plews N, Law M, Fysh A. Information transfer: what do decision makers want and need from researchers? Implementation Science 2007;2(20):1–12. doi: 10.1186/1748-5908-2-20 17608940 PMC1929120

[11] Pain R, Kesby M, Askins K. Geographies of impact: power, participation and potential. Area 2011;43(2):183–188.

[12] El-Jardali F, Ataya N, Fadlallah R. Changing roles of universities in the era of SDGs: rising up to the global challenge through institutionalising partnerships with governments and communities. Health Research Policy and Systems 2018;16(38). 10.1186/s12961-018-0318-9 PMC5944140 29743112

[13] Rabbani F, Shipton L, White F, Nuwayhid I, London L, Ghaffar A, et al. Schools of public health in low and middle-income countries: an imperative investment for improving the health of populations? BMC public health 2016;16(941):1–12. doi: 10.1186/s12889-016-3616-6 27604901 PMC5015344

[14] Doberstein C. The credibility chasm in policy research from academics, think tanks, and advocacy organizations. Canadian Public Policy 2017;43(4):363–375.

[15] Calavita K. Engaged Research, "Goose Bumps," and the Role of the Public Intellectual. Law and Society Review 2002;36(1):5–20.

[16] Van de Ven A H. Engaged Scholarship: A Guide for Organizational and Social Research. New York, USA: Oxford University Press Inc.; 2007.

[17] Bowen SJ, Graham ID. From knowledge translation to engaged scholarship: promoting research relevance and utilization. Archives of Physical Medicine and Rehabilitation 2013;94(1):S3–S8. doi: 10.1016/j.apmr.2012.04.037 23141502

[18] Calleson DC, Jordan C, Seifer SD. Community-engaged scholarship: Is faculty work in communities a true academic enterprise? Academic Medicine 2005;80(4):317–321. doi: 10.1097/00001888-200504000-00002 15793012

[19] Beaulieu M, Breton M, Brousselle A. Conceptualizing 20 years of engaged scholarship: A scoping review. PLoS One 2018;13(2). doi: 10.1371/journal.pone.0193201 29489870 PMC5831004

[20] Koon AD, Rao KD, Tran NT, Ghaffar A. Embedding health policy and systems research into decision-making processes in low-and middle-income countries. Health Research Policy and Systems 2013;11(30):1–9. doi: 10.1186/1478-4505-11-30 23924162 PMC3750690

[21] Olivier J, Scott V, Molosiwa D, Gilson L. Systems approaches in health systems research: approaches for embedding research. In: de Savigny D, Blanchet K, Adam T, editors. Applied Systems Thinking for Health Systems Research: A Methodological Handbook London: Open University Press, McGraw-Hill Education; 2017. p. 14–52.

[22] Johnston R, Plummer P. What is policy-oriented research? Environment and Planning A 2005;37(9):1521–1526.

[23] Oxford English Dictionary. English Dictionary. 2019; Available at: https://en.oxforddictionaries.com/. Accessed May, 2019.

[24] Merriam-Webster Dictionary. English Dictionary. 2019; Available at: https://www.merriam-webster.com/. Accessed May, 2019.

[25] National Conference of State Legislatures. How States Define Lobbying and Lobbyist. 2019; Available at: http://www.ncsl.org/research/ethics/50-state-chart-lobby-definitions.aspx.

[26] National Association of County and City Health Officials. Building Your Advocacy Tool Box: Advocacy vs. Lobbying. 2016.

[27] Gordon AK, Chung K, Handler A, Turnock BJ, Schieve LA, Ippoliti P. Final Report on Public Health Practice Linkages Between Schools of Public Health and State Health Agencies. Journal Of Public Health Management And Practice 1999;5(3):25–34. doi: 10.1097/00124784-199905000-00006 10537604

[28] Jessani NS, Babcock C, Siddiqi S, Davey-Rothwell M, Ho S, Holtgrave DR. Relationships between public health faculty and decision-makers at four governmental levels: A social network analysis. Evidence & Policy: A Journal of Research, Debate and Practice 2018;14(3):499–522.

[29] Jessani N, Kennedy C, Bennett SC. Enhancing evidence-informed decision-making: strategies for engagement between public health faculty and policymakers in Kenya. Evidence & Policy: A Journal of Research, Debate and Practice 2016;13(2):225–253.

[30] Schieve LA, Handler A, Gordon AK, Ippoliti P, Turnock BJ. Public health practice linkages between schools of public health and state health agencies: results from a three-year survey. Journal Of Public Health Management And Practice 1997;3(3):29–36. doi: 10.1097/00124784-199705000-00008 10186721

[31] Cairney P, Oliver K, Wellstead A. To bridge the divide between evidence and policy: reduce ambiguity as much as uncertainty. Public Administration Review 2016;76(3):399–402.

[32] Feldman M, Desrochers P. Research universities and local economic development: Lessons from the history of the Johns Hopkins University. Industry and Innovation 2003;10(1):5–24.

[33] Brownson RC, Kreuter MW, Arrington BA, True WR. Translating scientific discoveries into public health action: How can schools of public health move us forward? Public Health Reports 2006;121(1):97–103. doi: 10.1177/003335490612100118 16416704 PMC1497798

[34] Longest B Jr, Huber G. Schools of Public Health and the Health of the Public: Enhancing the Capabilities of Faculty to Be Influential in Policymaking. American Journal of Public Health 2010;100(1):49. doi: 10.2105/AJPH.2009.164749 19910342 PMC2791261

[35] Fraser I. Organizational research with impact: Working backwards. Worldviews on Evidence‐Based Nursing 2004;1(suppl_1):s52–s59. doi: 10.1111/j.1524-475X.2004.04044.x 17129335

[36] Mitton C, Adair CE, McKenzie E, Patten SB, Perry BW. Knowledge transfer and exchange: review and synthesis of the literature. Milbank Quarterly 2007;85(4):729. doi: 10.1111/j.1468-0009.2007.00506.x 18070335 PMC2690353

[37] Jacobson N, Butterill D, Goering P. Organizational factors that influence university-based researchers’ engagement in knowledge transfer activities. Science Communication 2004;25(3):246–259.

[38] McVay AB, Stamatakis KA, Jacobs JA, Tabak RG, Brownson RC. The role of researchers in disseminating evidence to public health practice settings: a cross-sectional study. Health Research Policy and Systems 2016;14(42):1–9. doi: 10.1186/s12961-016-0113-4 27282520 PMC4901476

[39] Stamatakis KA, Norton WE, Stirman SW, Melvin C, Brownson RC. Developing the next generation of dissemination and implementation researchers: insights from initial trainees. Implement Sci 2013 March 12;8:29–5908. doi: 10.1186/1748-5908-8-29 23497462 PMC3626831

[40] Wangenge-Ouma G, Lutomiah A, Langa P. Academic incentives for knowledge production in Africa. Case studies of Mozambique and Kenya. In: Cloete N, Maassen P, editors. Knowledge production and contradictory functions in African higher education. 1st ed.: African Minds; 2015. p. 124–147.

[41] Jessani NS, Valmeekanathan A, Babcock C, Ling B. Academic incentives for enhancing faculty engagement with decision-makers—considerations and recommendations from one School of Public Health. Humanities and Social Sciences Communications 2020;7(148).

[42] Kalbarczyk A, Rodriguez DC, Mahendradhata Y, Sarker M, Seme A, Majumdar P, et al. Barriers and facilitators to knowledge translation activities within academic institutions in low- and middle-income countries. Health Policy Plan 2021 June 03;36(5):728–739. doi: 10.1093/heapol/czaa188 33661285 PMC8173595

[43] Wye L, Cramer H, Carey J, Anthwal R, Rooney J, Robinson R, et al. Knowledge brokers or relationship brokers? The role of an embedded knowledge mobilisation team. Evidence & Policy: A Journal of Research, Debate and Practice 2018;15(2):277–292.

[44] Calignano G, Quarta CA. University of Salento’s Transactional Relations: Assessing the Knowledge Transfer of a Public University in Italy. Erdkunde 2014;68(2):109–123.

[45] Ross S, Lavis J, Rodriguez C, Woodside J, Denis JL. Partnership experiences: Involving decision-makers in the research process. J Health Serv Res Policy 2003 October 01;8(Suppl 2):26–34. doi: 10.1258/135581903322405144 14596745

[46] Stanley A, Zussman T. Strengthening networks and building relationships to increase the impact of global development research. 2016.

[47] Sin CH. The role of intermediaries in getting evidence into policy and practice: some useful lessons from examining consultancy–client relationships. Evidence & Policy: A Journal of Research, Debate and Practice 2008;4(1):85.

[48] Jessani NS, Valmeekanathan A, Babcock C, Ling B, Davey-Rothwell MA, Holtgrave DR. Exploring the evolution of engagement between academic public health researchers and decision-makers: from initiation to dissolution. Health Res Policy Syst 2020 February 10;18(1):15–019. doi: 10.1186/s12961-019-0516-0 32039731 PMC7011533

[49] Askins K. ‘That’s just what I do’: Placing emotion in academic activism. Emotion, Space and Society 2009;2(1):4–13.

[50] Chatterton P. Demand the possible: journeys in changing our world as a public activist-scholar. Antipode 2008;40(3):421–427.

[51] Roberts N, King P. Transforming Public Policy: Dynamics of Policy Entrepreneurship and Innovation. San Francisco, CA: Jossey-Bass; 1996.

[52] Johnson AT, Hirt JB, Hoba P. Higher education, policy networks, and policy entrepreneurship in Africa: the case of the Association of African Universities. Higher Education Policy 2011;24(1):85–102.

[53] Pain R. Social geography: seven deadly myths in policy research. Progress in Human Geography 2006;30(2):250–259.

[54] Peake L, Kobayashi A. Policies and practices for an anti-racist geography at the millennium. Professional Geographer 2002;54(1):50–61.

[55] Smith KE, Stewart EA. Academic advocacy in public health: Disciplinary ‘duty’ or political ‘propaganda’? Social Science & Medicine 2017;189(1):35–43. doi: 10.1016/j.socscimed.2017.07.014 28780438

[56] Blenner SR, Lang CM, Prelip ML. Shifting the culture around public health advocacy: Training future public health professionals to be effective agents of change. Health Promotion Practice 2017;18(6):785–788. doi: 10.1177/1524839917726764 28891348

[57] McAneney H, McCann JF, Prior L, Wilde J, Kee F. Translating evidence into practice: a shared priority in public health? Social science & medicine 2010;70(10):1492–1500. doi: 10.1016/j.socscimed.2010.01.038 20207462

[58] Doberstein C. Whom Do Bureaucrats Believe? A Randomized Controlled Experiment Testing Perceptions of Credibility of Policy Research. Policy Studies Journal 2017;45(2):384–405.

[59] Kotcher JE, Myers TA, Vraga EK, Stenhouse N, Maibach EW. Does engagement in advocacy hurt the credibility of scientists? Results from a randomized national survey experiment. Environmental Communication 2017;11(3):415–429.

[60] Yale School of Public Health. Activism and Advocacy. 2018; Available at: https://publichealth.yale.edu/about/gateways/students/MPH/MPH_academics/activism_advocacy.aspx. Accessed May, 2019.

[61] MacKenzie EJ. The Case for Advocacy. Hopkins Bloomberg Public Health Magazine 2018.

[62] Johns Hopkins Bloomberg School of Public Health. Center for Public Health Advocacy. 2019; Available at: https://www.jhsph.edu/research/centers-and-institutes/center-for-public-health-advocacy/index.html. Accessed May, 2019.

[63] Northeastern University, School of Law. Public Health Advocacy Institute. 2019; Available at: https://www.northeastern.edu/law/academics/institutes/phai.html. Accessed May, 2019.

[64] UCLA Fielding School of Public Health. The Public Health Training Program on Population Health Advocacy. 2013; Available at: https://ph.ucla.edu/current-students/public-health-training-program-population-health-advocacy.

[65] UC Berkley School of Public Health. Advocacy Initiative. 2018; Available at: http://sph.berkeley.edu/leadership-development/advocacy-initiative. Accessed May, 2019.

[66] Johns Hopkins Bloomberg School of Public Health. Certificate for Public Health Advocacy. 2019; Available at: https://www.jhsph.edu/academics/certificate-programs/certificates-for-hopkins-and-non-degree-students/public-health-advocacy.html. Accessed May, 2019.

[67] Johns Hopkins Bloomberg School of Public Health. Policy Advocacy in Low and Middle-Income Countries: Application for Real World Challenges. 2018; Available at: https://www.jhsph.edu/courses/course/27580/2019/221.633.11/policy-advocacy-in-low-and-middle-income-countries. Accessed May, 2019.

[68] Ayah R, Jessani N, Mafuta EM. Institutional capacity for health systems research in East and Central African schools of public health: knowledge translation and effective communication. Health Res Policy Syst 2014 June 02;12(1):20. doi: 10.1186/1478-4505-12-20 24890939 PMC4064507

[69] Spagnolo J, Gautier L, Champagne F, Leduc N, Melki W, N’Guessan K, et al. Reflecting on knowledge translation strategies from global health research projects in Tunisia and the Republic of Cote d’Ivoire. International journal of public health 2020 October 17. doi: 10.1007/s00038-020-01502-3 33068122

[70] Cole D, Jackson S, Forman L. What Approaches Can Schools of Public Health Take to Engage in Global Health? Reflections on the Implications of a Conceptual Synthesis. Global Health Governance 2017;11(2):71–83.

[71] Johns Hopkins University. Institutional Lobbying and Electoral Activity Frequently Asked Questions. 2015.

[72] Johns Hopkins University. Office of Government and Community Affairs. 2019; Available at: http://web.jhu.edu/administration/gca.

[73] Johns Hopkins Bloomberg School of Public Health. Health Advocacy. 2018; Available at: https://www.jhsph.edu/courses/course/27058/2018/301.645.01/health-advocacy. Accessed May, 2019.

[74] Johns Hopkins Bloomberg School of Public Health. Media Advocacy and Public Health: Theory and Practice. 2018; Available at: https://www.jhsph.edu/courses/course/27247/2018/410.663.01/media-advocacy-and-public-health-theory-and-practi. Accessed May, 2019.

[75] Pollack KM, Samuels A, Frattaroli S, Gielen A. The translation imperative: moving research into policy. Injury Prevention 2010;16:141–142. doi: 10.1136/ip.2010.026740 20363824

[76] McGinty EE, Frattaroli S, Appelbaum PS, Bonnie RJ, Grilley A, Horwitz J, et al. Using research evidence to reframe the policy debate around mental illness and guns: process and recommendations. Am J Public Health 2014 November 01;104(11):e22–6. doi: 10.2105/AJPH.2014.302171 25211757 PMC4202989

[77] Kromm JN, Frattaroli S, Vernick JS, Teret SP. Public Health Advocacy in the Courts: Opportunities for Public Health Professionals. Public Health Reports 2009;124(6):889–894. doi: 10.1177/003335490912400618 19894433 PMC2773956

[78] Hines A, Jernigan DH. Developing a comprehensive curriculum for public health advocacy. Health Promotion Practice 2012;13(6):733–737. doi: 10.1177/1524839912457682 22991279

[79] Jernigan DH. Meeting the challenge of change. Health Promotion Practice 2010;11(1):21–22. doi: 10.1177/1524839909354459 20038650

[80] Freudenber N. Public health advocacy to change corporate practices: implications for health education practice and research. Health Education & Behavior 2005;32(3):298–319. 15851541 10.1177/1090198105275044

[81] Bill & Melinda Gates Institute for Population and Reproductive Health. The Advance Family Planning (AFP) initiative. 2019; Available at: https://www.advancefamilyplanning.org.

[82] Rimon O, Fredrick B, Jernigan DH, Hinson L, Zuccaro J, JHSPH Advocacy Working Group. White Paper on Advocacy in Public Health. Invalid date Invalid date.

[83] Oliver K, de Vocht F, Money A, Everett M. Who runs public health? A mixed-methods study combining qualitative and network analyses. Journal of Public Health 2013;35(3):453–459. doi: 10.1093/pubmed/fdt039 23564840

[84] Contandriopoulos D, Benoît F, Bryant-Lukosius D, Carrier A, Carter N, Deber R, et al. Structural analysis of health-relevant policy-making information exchange networks in Canada. Implementation Science 2017;12(1):116. doi: 10.1186/s13012-017-0642-4 28931436 PMC5607603

[85] Jessani N, Kennedy C, Bennett SC. The Human Capital of Knowledge Brokers: An analysis of attributes, capacities and skills of academic teaching and research faculty at Kenyan schools of public health. Health Research Policy and Systems 2016;14(58). doi: 10.1186/s12961-016-0133-0 27484172 PMC4971650

[86] ATLAS ti Scientific Software Development GmbH. ATLAS.ti. 2017.

[87] Gale NK, Heath G, Cameron E, Rashid S, Redwood S. Using the framework method for the analysis of qualitative data in multi-disciplinary health research. BMC medical research methodology 2013;13(117):1–8. doi: 10.1186/1471-2288-13-117 24047204 PMC3848812

[88] Ruddick S. Activist geographies: building possible worlds. In: Cloke P, Crang P, Goodwin M, editors. Envisioning Human Geographies London: Arnold; 2004.

[89] Pain R. Social geography: on action orientated research. Progress in Human Geography 2003;27(5):649–657.

[90] Carlisle S. Health promotion, advocacy and health inequalities: a conceptual framework. Health Promotion International 2000;15(4):369–376.

[91] Cairney P, Oliver K. How should academics engage in policymaking to achieve impact? Political Studies Review 2018.

[92] Oliver K, Cairney P. The dos and don’ts of influencing policy: a systematic review of advice to academics. Palgrave Communications 2019;5(21):1–11.

[93] Kothari A, MacLean L, Edwards N. Increasing capacity for knowledge translation: understanding how some researchers engage policy makers. Evidence & Policy: A Journal of Research, Debate and Practice 2009;8(1):1–21.

[94] Boyko JA, Lavis JN, Abelson J, Dobbins M, Carter N. Deliberative dialogues as a mechanism for knowledge translation and exchange in health systems decision-making. Social Science & Medicine 2012;75(11):1938–1945. doi: 10.1016/j.socscimed.2012.06.016 22938912

[95] Lavis JN, Oxman AD, Lewin S, Fretheim A. SUPPORT Tools for evidence-informed health Policymaking (STP). Health Research Policy and Systems 2009;7(1). 10.1186/1478-4505-7-S1-I1 PMC3271819 20018098

[96] The SURE Collaboration. SURE Guides for Preparing and Using Evidence -Based Policy Briefs. 2011.

[97] American Association for the Advancement of Science. Science & Technology Policy Fellowships. 2019; Available at: https://www.aaas.org/programs/science-technology-policy-fellowships. Accessed June, 2019.

[98] Robert Wood Johnson Foundation. Health Policy Fellows. 2019; Available at: http://www.healthpolicyfellows.org/. Accessed June, 2019.

[99] Reicher S. COVID-19 has sparked new relationships between academia and policymakers–we must maintain them. 2021.

[100] Wesselink A, Buchanan k, Georgiadou Y, Turnhout E. Technical Knowledge, Discursive Spaces and Politics at the Science–Policy Interface. Environmental Science & Policy 2013;30:1–9.

[101] Weible CM, Ingold K. Why advocacy coalitions matter and practical insights about them. Policy & Politics 2018;46(2):325–343.

[102] McGinty EE, Siddiqi S, Linden S, Horwitz J, Frattaroli S. Improving the use of evidence in public health policy development, enactment and implementation: a multiple-case study. Health Education Research 2019;34(2):129–144. doi: 10.1093/her/cyy050 30601978

[103] Cvitanovic C, Cunningham R, Dowd AM, Howden SM, Putten EI. Using social network analysis to monitor and assess the effectiveness of knowledge brokers at connecting scientists and decision‐makers: An Australian case study. Environmental Policy and Governance 2017;27(3):256–269.

[104] Kislov R, Wilson PM, Knowles S, Boaden R. Learning from the emergence of NIHR Collaborations for Leadership in Applied Health Research and Care (CLAHRCs): a systematic review of evaluations. Implementation Science 2018;13(1):111. doi: 10.1186/s13012-018-0805-y 30111339 PMC6094566

[105] World Health Organization, Evidence-Informed Policy Network. EVIPNet in action: 10 years, 10 stories. 2016.

[106] El-Jardali F, Ataya N, Jamal D, Jaafar M. A multi-faceted approach to promote knowledge translation platforms in eastern Mediterranean countries: climate for evidence-informed policy. Health Research Policy and Systems 2012;10(1):15. doi: 10.1186/1478-4505-10-15 22559007 PMC3445832

[107] Maxey I. Beyond boundaries? Activism, academia, reflexivity and research. Area 1999;31(3):199–208.

[108] MacGregor H, Bloom G. Health systems research in a complex and rapidly changing context: ethical implications of major health systems change at scale. Developing world bioethics 2016;16(3):158–167. doi: 10.1111/dewb.12115 26957047 PMC5111606

[109] Biesta G. Why “what works” won’t work: Evidence‐based practice and the democratic deficit in educational research. Educational Theory 2007;57(1):1–22.

[110] Lin V, Gibson B. Evidence-based health policy: problems and possibilities. Oxford, UK: Oxford University Press; 2003.

[111] Marston G, Watts R. Tampering with the evidence: a critical appraisal of evidence-based policy-making. The drawing board: An Australian review of public affairs 2003;3(3):143–163.

[112] Neylan J. Social policy and the authority of evidence. Australian Journal of Public Administration 2008;67(1):12–19.

[113] Triantafillou P. The political implications of performance management and evidence-based policymaking. The American Review of Public Administration 2015;45(2):167–181.

[114] Bolder Advocacy: A Program of Justice and Alliance. Enabling nonprofits to shape the public debate on important social issues. 2019; Available at: https://bolderadvocacy.org/resource-library/.

[115] American College of Surgeons. Advocacy, Lobbying, and Political Activities. 2019; Available at: https://www.facs.org/member-services/chapters/chapter-guidebook/advocacy#viewport.

[116] Johns Hopkins Bloomberg School of Public Health. The Power of Public Health: A Strategic Plan for the Future FY2019-2023. 2018.

[117] MacKenzie EJ, Klag MJ, Sommer A. The Bloomberg American Health Initiative. Public Health Reports 2018;133(1_suppl):5S–8S. doi: 10.1177/0033354918798375 30426877 PMC6243445

[118] Sharfstein JM, Leighton J, Sommer A, MacKenzie EJ. Public Health Rising to the Challenge: The Bloomberg American Health Initiative. Public Health Reports 2018;133(1_suppl):3S–4S. doi: 10.1177/0033354918799744 30426869 PMC6243449

