# Supplementary material for: Advocacy, activism, and lobbying: How variations in interpretation affects ability for academia to engage with public policy
Source: PLOS Glob Public Health. 2022 Mar 18;2(3):e0000034. doi: 10.1371/journal.pgph.0000034 (PMC10021895; doi:10.1371/journal.pgph.0000034)
Supplement: S1 Table — (DOCX) [file pgph.0000034.s001.docx]

S1 Table: British and American Dictionary Definitions of Terms

| Dictionary Term | Oxford English Dictionary (EOD) Definition | Merriam Webster Dictionary (MWD) Definition |
| --- | --- | --- |
| Advocate (n) | 1. A person who publicly supports or recommends a particular cause or policy 2. A person who puts a case on someone else's behalf | 1. One who pleads the cause of another 2. One who defends or maintains a cause or proposal 3. One who supports or promotes the interests of a cause or group |
| Advocate (v) | To publicly recommend or support | To support or argue for (a cause, policy, etc.) |
| Activist (n) | A person who campaigns to bring about political or social change | A person who uses or supports strong actions (such as public protests) in support of or opposition to one side of a controversial issue |
| Activism (v) | The policy or action of using vigorous campaigning to bring about political or social change | A doctrine or practice that emphasizes direct vigorous action especially in support of or opposition to one side of a controversial issue |
| Lobbyist (n) | A person who takes part in an organized attempt to influence legislators | 1. One who conducts activities aimed at influencing or swaying public officials and especially members of a legislative body on legislation 2. A person engaged in lobbying public officials |
| Lobby (v) | To Seek to influence (a legislator) on an issue | 1. To conduct activities aimed at influencing public officials and especially members of a legislative body on legislation 2. To attempt to influence or sway (someone, such as a public official) toward a desired action |
