# Supplementary material for: Advocacy, activism, and lobbying: How variations in interpretation affects ability for academia to engage with public policy
Source: PLOS Glob Public Health. 2022 Mar 18;2(3):e0000034. doi: 10.1371/journal.pgph.0000034 (PMC10021895; doi:10.1371/journal.pgph.0000034)
Supplement: S2 Table — (DOCX) [file pgph.0000034.s002.docx]

S2 Table: Faculty participant overview

| Characteristic | | Number | % of Total |
| --- | --- | --- | --- |
| Sex | M | 20 | 38% |
|  | F | 32 | 62% |
| Track | **Professorial** | 25 | 48% |
|  | Senior (Professor Emeritus, Professor, Associate Professor) | 21 | 40% |
|  | Junior (Assistant Professor, Lecturer) | 4 | 8% |
|  | **Scientist** | 24 | 46% |
|  | Senior (Senior Scientist, Associate Scientist) | 5 | 10% |
|  | Junior (Assistant Scientist, Snr. Research associate, Research associate) | 19 | 36% |
|  | **Other** | 3 | 6% |
| Leadership positions | Yes | 15 | 29% |
|  | No | 37 | 71% |
