# Supplementary material for: Advocacy, activism, and lobbying: How variations in interpretation affects ability for academia to engage with public policy
Source: PLOS Glob Public Health. 2022 Mar 18;2(3):e0000034. doi: 10.1371/journal.pgph.0000034 (PMC10021895; doi:10.1371/journal.pgph.0000034)
Supplement: S1 File — (PDF) [file pgph.0000034.s003.pdf]

Oral Consent Script 1_Int'l Min Risk
DO NOT USE TO ENROLL PARTICIPANTS
(Once approved, IRB logo goes here)

Approval date:
Approved consent IRB version No.:

INTERVIEW GUIDE FOR ACADEMIC FACULTY

Study Title: The influence of JHSPH faculty on public health decision-making: A mixed methods study exploring networks, relationships and engagement strategies
IRB No.: IRB00006968
PI Version Date: V3 2 November 2017

UNIQUE ID:__________

SECTION 1: DEMOGRAPHIC AND SOCIOECONOMIC INFORMATION

This section will have already completed during phase I of the study involving sociometric surveys for those faculty who participated in phase I. If the IDI is with faculty who did not partake in Phase I then rephrase 1.1 and 1.2 to capture the new information

1.1) Please let me know if there have been any changes in the following demographic characteristics since we last spoke (*mention date of survey in the event that the respondent cant recall)*:

- 1a. Primary department at JHSPH
- 1b. Academic position
- 1c. Leadership or administrative position

1.2) In the previous interview you mentioned that you were [currently engaged with decision-makers at the City, State, Federal, and Global] level] or [currently not engaged with decision-makers]. Has this changed in any way? (Probe for more details about their current engagement/non engagement).

SECTION 2: ROLE OF RESEARCHERS IN BRINGING EVIDENCE TO BEAR ON DECISION-MAKING

(*The questions in this section are general questions that will help set the scene for questions later and provides background on the respondent's understanding of the decision-making process as well as their perceptions about the role of researchers without putting them on the spot about their own engagement which comes in section 3A or 3B)*

We will now explore the role of researchers in bringing evidence to bear on decision-making. The next few questions focus on why faculty do engage with decision-makers, why some may not, and the institutional environment that influences this.

2.1) What are your thoughts on why academic faculty at JHSPH engage with the decision-making process? *Describe what we mean by the DM process so that all respondents approach the question the same way)*

- *probe: both direct and indirect activities*
- *Probe: reasons for engagement.*

2.2) What are your thoughts on why some academic faculty at JHSPH do not engage with the decision-making process?

- *probe: organizational constraints, personal capacities, values, comfort, knowledge etc…)*

2.3) How would you describe the current institutional environment with respect to academic faculty engaging with decision makers?

- *(Probe: culture, values, policies, ... if these are implicit, explicit and how they are shared. If vague ask respondent for examples)*
- Has this changed over time? In what way?

2.4) In your experience, how has working with decision-makers affected the career advancement opportunities for you or your peers at the SPH? *(Probe incentives and disincentives, ask for examples of situations where each has occurred)*

a) Are there written and/or unwritten guidelines about faculty engagement with decision-makers in the A & P process?
   a. *Probe more details about these guidelines, <u>If need be rephrase to:</u> to what level if any is policy engagement valued by your dept)*
   b. *Probe: If the respondent replies with "I don't know", provide a diplomatic probe to understand why*

b) How does experience in working with decision-makers affect faculty recognition within JHSPH beyond the A & P process? *(Probe incentives and disincentives, ask for examples of situations where each has occurred)*

IF RESPONDENT INDICATED STILL ENGAGING IN EVIDENCE TO DECISION-MAKING ACTIVITIES IN SECTION 1 Q2, GO TO 3A
IF RESPONDENT INDICATED NOT ENGAGING IN EVIDENCE TO POLICY ACTIVITIES IN SECTION 1 Q2, GO TO 3B
IF RESPONDENT INDICATED BEING IN AN ACADEMIC LEADERSHIP ROLE IN SOCIOMETRIC SURVEY OR IN SECTION 1 Q1, ADMINISTER 3C IN ADDITION TO EITHER 3A OR 3B

SECTION 3A: ENGAGED FACULTY [SKIPPED FOR PHASE 1 PARTICIPANTS WHO REPORTED NO ENGAGEMENT]

In the previous survey, we explored all the ways in which you engage with decision makers. I will quickly summarize those for you here. Please let me know if I missed anything *(use survey to remind respondent of what they had said)*

3A.1) On a scale of 1-5 (5 being the highest), how in-depth or detailed do you feel your understanding of how health policies and programs are made [city, state, federal or global level]?

a) Could you perhaps help me understand the process form your experiences at any of these levels?
   Probe: What is the process? Who are the key players? What are the challenges? What affects evidence use? How does it vary at these various levels?

3A.2) In your experience, what kind of research or evidence is MOST relevant and useful for decision-makers?
(Probe: *Disciplinary area? Methodological preferences? Cost-effectiveness data? Etc…)*

- In what form and format do they prefer to receive it?
- Do you feel that you are able to provide it in the desired format? Why/Why not?

3A.3) Please provide an overview of the nature of your involvement with decision-makers at any of the 4 government levels we focused on? *(This question aims to provide a sense of the types of exchanges that the informant has had and that can be used in the rest of the interview. Probe: what are the different ways in which you get involved? At what level? At what point in the process? How does it begin – active pursuit or by invitation? How long have you been involved?)*

a. Please walk me through one particular example of your involvement in a policy issue?
b. Probe: How did you become involved in this issue?
c. Probe: was this engagement intentional or serendipitous? Who initiated? Why? At what point in the process? How did it evolve? How did it end?
d. Probe: What was the role of research evidence in the decision-making discussions?
e. Probe: Were you the only researcher involved or were there others?
f. Probe: Can you tell me about the evidence that was used to inform the policy options?*(eg: specific publications, studies, reviews)*
g. Probe: Can you tell me how the evidence came to be shared with decision-makers?
h. Probe: What outcomes or impact did this engagement lead to?

3A.4) Why do you personally engage in such activities? (*This question aims to understand the motivations behind E2P engagement)*

3A.5) Given your involvement with decision-makers, could you perhaps share what factors are the MOST IMPORTANT in affecting the creation and maintenance of these **relationships**? *(Probe: facilitators, barriers)*

3A.6) How/In what way do you encourage and support **decision-makers** to **use research evidence in their decision making and policies**? *(This question aims to provide a sense of the types of strategies that the informant employs when sharing research results with decision makers. As the respondent is speaking, Interviewer will construct a list of strategies based on their narrative. Interviewer will use this list to guide a discussion related to process, people, events, and outcomes).*

	Strategy [A]	[Strategy B]	[Strategy C]	[Strategy D]	[Strategy E]
a. When is such a strategy effective? (in what situations?)					
b. Why is this strategy effective in such a situation?					

(If worried about time, skip 3A.7 and 3A.8 and focus on 3A.9)

3A.7) In Phase 1 of this study, faculty reported that the key factors that facilitated decision-making engagement. I am going to list the top 3 facilitators reported, please let me know your thoughts on this list (*probe into whether they are surprised? Whether they think it is dependent on department etc…*).

a. Introductions to decision-makers by colleagues with relevant relationships
b. Being affiliated with JHSPH
c. Relevance of your research to pertinent policy issues

3A.8) In Phase 1 of this study, faculty reported that the key factors that hindered their decision-making engagement. I am going to list the top 3 barriers reported, please let me know your thoughts on this list. (*probe into whether they are surprised? Whether they think it is dependent on department etc…*).

a. Time for engagement
b. Geographic location of your workplace

c. Your stage of career

3A.9) In Phase 1 of the study, faculty reported JHSPH leadership should focus on these 3 priorities to further promote faculty engagement with decision-makers:

a. Create academic incentives
b. provide supplemental internal funding for activities
c. create a culture of pursuing policy-relevant research.

What are your thoughts on these priorities? Would you list a different priority?

3A.10) If a colleague approached you to ask you what s/he could do to be more active in *engaging with decision-making* (agenda setting, policy formulation etc.), what advice would you give them?

SECTION 3B: FACULTY NOT ACTIVELY ENGAGED WITH DECISION MAKERS

3B.1) We know that there are many reasons why faculty do not engage with decision makers. What are some of the reasons for you personally*?*

(Probe: skills, incentives, resources, motivation etc)

a. *If respondent was previously but not currently engaged: What factors led to the dissolution of previous relationships with decision-makers.*

3B.2) Have you shared or promoted your research with any non-academic stakeholders?

(*Probe: Who, how, why? Doesn't have to be decision-makers…could be NGOs, advocacy organisations)*

3B.3) In Phase 1 of this study, faculty reported that the key factors that facilitated decision-making engagement. I am going to list the top 3 facilitators reported, please let me know your thoughts on this list (*probe into whether they are surprised? Whether they think it is dependent on department etc…*).

a. Introductions to decision-makers by colleagues with relevant relationships
b. Being affiliated with JHSPH
c. Relevance of your research to pertinent policy issues

3B.4) In Phase 1 of this study, faculty reported that the key factors that hindered their decision-making engagement. I am going to list the top 3 barriers reported, please let me know your thoughts on this list. (*Probe into whether they are surprised? Whether they think it is dependent on department etc…*)

a. Time for engagement
b. Geographic location of your workplace
c. Your stage of career

3B.5) In Phase 1 of the study, faculty reported JHSPH leadership should focus on these 3 priorities to further promote faculty engagement with decision-makers:

a. Create academic incentives
b. provide supplemental internal funding for activities
c. create a culture of pursuing policy-relevant research.

What are your thoughts on these priorities? Would you list a different priority?

SECTION 4: ORGANIZATIONAL OFFERINGS

JHSPH has been offering several seminars, capacity building workshops, etc…in this area.

4.1) If JHSPH were to offer more training to faculty and students on engaging with decision makers – in what areas do you think the SPH should focus?

- What would be the 3-5 most important things the SPH could be doing/offering? *(EG: how to write op-eds, how to present without jargon, how to prepare for meetings, how to build a network of relations, how to include CEA in policy briefs? Etc)*
- To what extent do you think that the SPH is addressing these needs?

4.2) Here is a list of JHSPH activities that we are aware of. *(Provide list of all relevant activities that we have compiled for this question)* Have you been able to attend or access any of these?

a. If yes, which ones and what are your reflections on them? *(Probe: how did you hear about them? have they been useful, have you used any of the information to affect your won work? If so, how?)*
b. If no, why not?

c. Do you access the SPH's support for advocacy/lobbying services?

SECTION 5: CLOSURE

I don't have any more questions for you but is there anything that you would like to share with me about any of the topics we covered above or that you think would be important for me to know?

Do you have any questions for me?

That's the end of our interview today. Thank you very much for your time. Would you mind if I contacted you again should I need any clarifications on this interview?
