# Supplementary material for: Advocacy, activism, and lobbying: How variations in interpretation affects ability for academia to engage with public policy
Source: PLOS Glob Public Health. 2022 Mar 18;2(3):e0000034. doi: 10.1371/journal.pgph.0000034 (PMC10021895; doi:10.1371/journal.pgph.0000034)
Supplement: S2 File — (PDF) [file pgph.0000034.s004.pdf]

Oral Consent Script 1_Int'l Min Risk<br>**DO NOT USE TO ENROLL PARTICIPANTS**<br>*(Once approved, IRB logo goes here)*	Approval date:<br>Approved consent IRB version No.:

IN-DEPTH INTERVIEW GUIDE FOR DECISION MAKER

Study Title: The influence of JHSPH faculty on public health decision-making: A mixed methods study exploring networks, relationships and engagement strategies
IRB No.: IRB00006968
PI Version Date: V3 2 Nov 2017

UNIQUE IDENTIFIER: ________________________

SECTION 1: ROLE OF RESEARCH IN DECISION MAKING

1.1) In your opinion, on a scale of 1-10 (10 being highest) where do you think research evidence lies with respect to informing decision making at your organization currently? *(Probe: Ask for the rationale for giving this rating)*

- Has this changed over time? If so, where on the scale would you rate its use in previous years?
- What are your thoughts on these changes? Why the change?

1.2) Does your organization currently conduct and/or commission research?

- Has this changed over the years? If so, in what way (increased or decreased?) *(Probe more on the response…see if we can elicit any trends and the reasons why)*

  Probe: If commissioned, how do you commission it? Open calls versus invitations? If invitations, how are experts identified?
  Probe: Could you provide an example of when you reached out to an academic researcher for a study and why?
  Probe: Please tell me a little bit of the internal research that is conducted at your site. Are academic faculty ever engaged in these project?

1.3) In what way(s) **are you currently engaged** with academic (medical or public) health researchers when making policy decisions of programmatic decisions?

- *Probe: How do you engage with researchers?*
- *Probe: are there formal mechanisms? Informal ones?*

1.4) What strategies do you personally use to **access and use research results** in your *own organization* for decision making? *(This question aims to provide a sense of the types of strategies that the informant employs for access and uptake for research results. As the*

respondent is speaking, study team member will construct a list of strategies based on their narrative. Interviewer will have a list handy to probe if needed).

	STRATEGY				
	A	B	C	D	E
a. When is such a strategy effective? (in what situations?)					
b. *What* makes this strategy effective in such a situation?					

- *Follow up: Do you suspect these strategies vary across the organization?*
  - *Probe: In what ways?*

1.5) What do you do to ensure academic organizations are responding to your needs and therefore aware of and involved in **identifying research interests and priorities**? *(This question aims to provide a sense of the types of strategies that the informant employs in communicating government (research) priorities. As the respondent is speaking, Interviewer will construct a list of strategies based on their narrative. Interviewer will use this list to guide a discussion related to process, people, events, and outcomes).*

	STRATEGY				
	A	B	C	D	E
a. When is such a strategy effective? (in what situations?)					
b. What makes this strategy effective in such a situation?					

Probe: Financial strategies, political, relational etc…

1.6) What steps do you take to ensure scientific integrity and ethics of the scientific community? (*How do you ensure investigative freedom? How do you manage potential conflicts of interest?)*

SECTION 2: ROLE OF RESEARCHERS IN BRINGING EVIDENCE TO BEAR IN DECISION-MAKING

2.1) In your experience, what are some of the reasons that decision-makers choose to engage with academic faculty?

a. What factors facilitate and hinder a long-standing relationship?

2.3) Could you share your thoughts on how you feel about academic faculty engaging with decision-makers in order to inform decision-making?

2.3) In your opinion, what is the most important thing about working with an academic partner? (Probe: *Does it depend on the individual? Reputation? Existing relationships? Etc…)*

2.4) Previously, you shared your general thoughts about engaging with academic faculty in decision-making. What are your thoughts about engaging JHSPH faculty in particular, in the decision-making process? *(If they are uncomfortable with this, then skip)*

a. *Probe: what are the reasons for why you engage with JHSPH faculty?*
b. *Probe: What spurred the relationships with these individuals (and how have they evolved)? (ie the affiliation or prior relations? Etc)*
c. *Probe: What have been your experiences with engagement with academic faculty in general? With JHSPH in particular?*

2.5) Could you walk me through one particular example of your **involvement with an academic faculty/researcher?** *((How did you become involved with them? Who initiated? Why? At what point in the process? How did it evolve? How did it end? What was the effect? Impact? Utility?)*

2.6) Could you walk me through one particular example of when you (or your colleagues) **used evidence to inform a decision or policy**?

a. Probe: What was the role of research evidence in the policy discussions?
b. Probe: Can you tell me about the types of evidence that were used to inform the policy options? *(Probe: specific publications, studies, reviews and how each was perceived: quality, relevance, local applicability)*
c. Probe: Can you tell me how the evidence came to be shared with you? *(This question aims to extract the methods and channels used to gather the necessary information)*

SECTION 3: PERSONAL AND ENVIRONMENTAL FACTORS *(IF RUNNING OUT OF TIME SKIP TO SECTION 4)*

We are interested in better understanding the factors – both individual as well as institutional/environmental - that either help or hinder researcher engagement with decision-makers and vice versa. I would like to understand your thoughts on some of these.

Decision-maker attributes

There have been several studies exploring the facilitators and barriers to engagement between researchers and decision makers. Here is a compiled list. *(Provide list of factors already identified in the literature)*

3.1) When looking at this list, in your experience, what *individual* characteristics or capacities do you think facilitates decision maker engagement with academics?

a. Probe: personality traits, skills, experiences, academic training, practical training
b. Probe: why they chose these? Can they give examples?

Environmental characteristics

3.2) *(Provide list of factors already identified in the literature)*

When looking at this list, in your experience, what *institutional or internal* factors of your organization resonate or are different?

A) Which ones facilitate engagement with academic faculty? Why/Say more?
B) Which ones hinder engagement with academic faculty? Why/Say more?

3.3) *(Provide list of factors already identified in the literature)*

When looking at this list, in your experience, what *external* factors affect evidence informed decision making?

a. Probe: environmental, political, social, Organizational, professional. Ask for specific examples of facilitators and of barriers

3.4) Now, I am going to ask you some questions about all of the barriers you just mentioned. In your opinion, which of these barriers should be a priority for alleviation? *(Probe: Why? How?)*

3.5) We have discussed several barriers already. What strategies have you or your colleagues used to overcome some of these barriers we have talked about? *(Probe about initiatives within the organization that have conducted to increase engagement with faculty (i.e trainings, partnerships, program to increase evidence use).*

	STRATEGY				
	A	B	C	D	E
a. When is such a strategy effective? (in what situations?)					
b. What makes this strategy effective in such a situation?					

SECTION 4: ADVICE

4.1) If a *researcher* approached you to ask you what s/he could do to engage in more policy influence, what advice would you give them (ie how do you think they should engage with persons such as yourself)?

4.2) If a decision-making *colleague* approached you to ask you what s/he could do to share policy/decision relevant priorities and processes with academic researchers, what advice would you give them?

4.3) If a *School of Public Health* wanted to enhance the capacity of faculty in engaging in research–to-policy activities, what would you advise them to do?

a. If JHSPH were to offer more training to faculty and students on engaging with decision makers – in what areas do you think the SPH should focus? *Remind them of the list in section 2 Q4 if that helps (EG: how to write op-eds, how to present without jargon, how to prepare for meetings, how to build a network of relations, how to include CEA in policy briefs? Etc)*

SECTION 5: DEMOGRAPHIC AND SOCIOECONOMIC INFORMATION

**UNIQUE IDENTIFIER**	
**5.1** Organization name	
**5.2** Years with Organization	
**5.3** Position	
**5.4** Years in current position	
**5.5** Sex	0= Male 1= Female 2=Other 3= prefer not to answer
**5.6** Age	1=<30<br>2= 30-34

	3= 35-39<br>4= 40-44<br>5= 45-49<br>6= 50-54<br>7= 55-59<br>8= 60-64<br>9= 65-69<br>10= 70-74<br>11=75-79<br>12 >=80
**5.7** Highest academic qualification obtained	1= University or college diploma;
	2= Bachelors degree
	3= Masters degree
	4= Doctoral degree (eg PhD) or medical degree;
	5= Post Doctoral fellowship

SECTION 6: CLOSURE

I don't have any more questions for you but is there anything that you would like to share with me about any of the topics we covered above or that you think would be important for me to know?

Do you have any questions for me?

That's the end of our interview today. Thank you very much for your time. Would you mind if I contacted you again should I need any clarifications on this interview?
